# How the COVID-19 Pandemic Changed Adolescents’ Use of Technologies, Sense of Community, and Loneliness: A Retrospective Perception Analysis

**DOI:** 10.3390/bs12070228

**Published:** 2022-07-13

**Authors:** Andrea Guazzini, Andrea Pesce, Fabiana Gino, Mirko Duradoni

**Affiliations:** 1 Department of Education, Literatures, Intercultural Studies, Languages and Psychology, University of Florence, 50135 Florence, Italy; andrea.guazzini@unifi.it (A.G.); andrea.pesce@stud.unifi.it (A.P.); fabiana.gino.psy@gmail.com (F.G.); 2 Centre for the Study of Complex Dynamics, University of Florence, 50019 Sesto Fiorentino, Italy

**Keywords:** remote learning, COVID-19, ICT, sense of community, school, loneliness

## Abstract

**Highlights:**

- The pandemic has massively exacerbated the sense of loneliness of high-school students, especially of young women.
- The pandemic has changed the use of technology by high-school students for social, information, leisure, and educational purposes.
- Young women changed their use of technology more than young men to stay in touch with their family and for information seeking and study purposes.
- High-school students’ sense of community has undergone modest variations due to the pandemic.

**Abstract:**

The COVID-19 pandemic has brought important changes to how we engage in relationships of any kind. To combat the spread of the virus, schools resorted to remote-learning, and teenagers had to rely on various technologies to meet many of the needs that they used to satisfy offline (e.g., social, informational, and recreational/leisure purposes). This article was written to investigate the changes that the students at an Italian high school went through in terms of use of technologies, loneliness, and sense of community, through a survey focusing on their retrospective perceptions. The study was carried out on 917 students. In general, we have found that the COVID-19 pandemic has greatly increased the perception of loneliness in teenagers (especially in female respondents), as well as their use of technologies for social, informational, and leisure purposes. However, maybe thanks to the opportunities provided by ICTs and remote learning, the sense of community in Italian teenagers was only marginally impacted.

## 1. Introduction

It has been more than 1 year since 11 March 2020 when the World Health Organization declared the COVID-19 virus a global pandemic. During this period, people have experienced unprecedented interruptions to their daily lives, including, in most cases, their work [1]. Children and adolescents experienced some critical moments too: in addition to the global fear felt for this new disease, they found themselves no longer having sociality, sports, and hobbies [2]. The pandemic had a strong impact on adolescents’ well-being in terms of mental illness, anxiety, depression, and stress-related symptoms [3,4], as well as perceived loneliness [5,6].

One of the environments most affected by the COVID-19 situation was the school. Data from UNESCO show that in April 2020, at least 192 countries had their schools closed, and almost 90% of elementary, middle, and high school students worldwide were affected by this situation: one year later, in April 2021, more than 177 million students were still affected, and schools are in many cases still either completely closed or only partially opened [7].

To avoid the risk of a total and prolonged interruption of the school year, and to guarantee the fulfillment of social distancing policies that are difficult to enforce in a class or in a school, in many cases it was decided to resort to online learning. Fry [8] defines online learning as the “delivery of training and education via networked interactivity and a range of other knowledge collection and distribution technologies”, and it can be asynchronous—i.e., the simultaneous participation of both students and teachers is not required—and synchronous, supported by video conferences that allow a real-time connection [9]. Institutions have been talking about this technology for many years, but it was apparently only considered as a valid alternative to traditional learning because of the COVID-19 emergency.

This type of learning has several advantages: it is flexible, interactive, and allows for self-regulation [10]; it has the benefit of reducing, if not eliminating, time and money spent on commuting; it makes it easy to access study material and to record lectures from home [11,12]; and it facilitates the discussion and sharing of ideas among students in asynchronous contexts [13]. However, there are also many points of criticism: on one side, students have expressed doubts about this practice, denouncing technical problems, a feeling of being isolated, and struggling with a lack of self-discipline [12,14]; on the other, it should be noted that there are several possible obstacles—for example, socio-economic factors, digital expertise, disturbances coming from the domestic environment, and, in many cases, an increased workload [10]. The transition from face-to-face lessons to online classes has indeed caught many teachers unprepared with considerable repercussions on the students [15]. In general, adolescents with traditional face-to-face relationships develop a sense of belonging to the school and their class. Alienation may be a major reason for students’ lack of sense of belonging to school [16]. During the pandemic, students were usually forced to attend lessons inside their room and no longer see their friends face-to-face, thus experiencing in some cases a significant sense of alienation [17].

Therefore, the limited opportunity for face-to-face socialization due to the pandemic could have decreased the quality and quantity of social relationships, thwarted belonging, and ultimately increased loneliness [18].

Sense of Community (SOC) has been described by McMillan and Chavis [19] as (a) a feeling that members have of belonging, (b) a feeling that members matter to one another and to the group, and (c) a shared faith that members’ needs will be met through their commitment to being together. Hill [20] concluded that the psychological sense of community refers to variables beyond individual relationships, that it appears to be setting specific, and that aspects of the concept differ from setting to setting. One such setting is the school, in particular the classroom, which was badly affected by the pandemic because, in many countries, regular classes were interrupted and, instead, remote learning and other compensating measures were introduced [7]. Nonetheless, in line with Social Identity Theory (SIT; [21]) and Social Categorization Theory (SCT; [22,23]), social categorization and identification processes already took place in the school and class environments and so these appraisal processes are stable if opportunities to socially engage are provided [24]. 

Teenagers are digital natives and so, to make up for the lack of face-to-face interactions, they increased their use of ICT, something that was already high. The way in which we approach technology is constantly changing, and the same could be said for the kind of technology adolescents use. According to Anderson and Jiang’s study [25], 95% of adolescents from the United States own a smartphone, and 45% have almost constant acces to the internet. In general, there has been an observable increase in the use of digital media: while television and printed press as media are declining among adolescents, the use of new technologies (i.e., messaging, Internet usage, and online gaming) has remained constantly high in recent years and, in particular, there has been an increase in the use of social networking sites that, especially for girls, have become an integral part of the daily routine for a high number of teenagers [26]. Girls also tend to use technology for social networking sites in a more consistent way than boys—especially those that are mostly focused on sharing multimedia content (i.e., Instagram, Snapchat)—while boys seem to mostly be involved in online gaming platforms or communities focused on specific interests [27]. However, it was found that in both genders, social media sites are seen as an important resource to interact with friends, find new friendships or receive support and advice, and interact with the community, but also to study, acquire new skills, and create connections that might be useful for their future academic career [27,28]. Within this framework, the COVID-19 pandemic ended up facilitating a further increase in ICT use [29], and now many teenagers are even exhibiting behavior that is very close to technology-addiction behavior [30]. People’s general sense of belonging is derived from, and dependent on multiple social identities at the same time [31], and ICT-based relationships may not be sufficient or adequate to fully compensate for all the lost face-to-face interactions that resulted from the pandemic in terms of both social and emotional loneliness [18]. Therefore, in line with the Need to Belong Theory and its latest developments [32,33,34], loneliness has been observed to have severely increased during the pandemic [35], despite the ICT support in helping adolescents and young adults maintain and safeguard some group membership and identification (e.g., school, class, close friends). 

### Study’s Aim and Hypothesis Development 

A number of studies have shown how the use of new technologies increased during the pandemic [36], especially in younger people [37]. For instance, the literature stresses that there was an increase in screen time in many different population groups across the globe during the COVID-19 outbreak [38], but this increased time is not equally distributed among every available activity (e.g., recreational, social, informational activities). Therefore, with this paper, the authors want to investigate in more detail the motivations that are tied to increased use of ICT during the COVID-19 pandemic. The scientific literature stresses that informational [39,40,41], social [42,43,44], and recreational motives [45,46,47] appeared to be some of the most important motivations in shaping people’s ICTs use [48]. Notably, no study so far has examined all these motivations at the same time in the current pandemic scenario, especially in adolescents, who usually make extensive use of ICTs [25].

Moreover, several studies have investigated how the pandemic and “forced” remote learning had an effect on variables like loneliness [5,6,49] and sense of community [13]; however, currently, there are no studies investigating these effects—specifically in the Italian teenage population. Overall, this work aims to fill the gaps that are currently present in the literature, by retrospectively investigating how ICT usage, sense of community, and loneliness perception changed in teenagers due to the COVID-19 pandemic and thus contribute to the discussion about the effects of remote learning on specific communities such as the community of teenage students. 

The pandemic and remote learning changed the use of adolescent technology to study [50], for social reasons (to keep in touch with their classes, their friends, their families, and to manage their social networks) and to stay updated on the news [40,43], and to entertain themselves [45,51]. Based on these findings, we expected that:

**H1.1.**  *There was an increase in the use of new technologies for study-related reasons.*

**H1.2.**  *There was an increase in the use of new technologies for social reasons.*

**H1.3.**  *There was an increase in the use of new technologies for informative reasons.*

**H1.4.**  *there was an increase in the use of new technologies for leisure reasons.*

The use of new technologies seemed to be affected by gender. In particular, young women referred to the use of ICT for social aims both pre-pandemic [52,53,54] and in the current period [5,14] more than young men, who seemed to use ICT for recreational reasons more than girls [42,55]. Before COVID-19, young men also were observed to use ICT for information-seeking behaviors more than young women [53]. For these reasons, in our study we expected the following to happen:

**H2.0.**  *The use of technology is influenced by gender.*

**H2.1.**  *Girls use technologies for ICT for social reasons more than boys do, both before and during the pandemic.*

**H2.2.**  *Boys use ICT for leisure more than girls do, both before and during the pandemic.*

**H2.3.**  *Boys use ICT for informative reasons more than girls do, both before and during the pandemic.*

Several works have highlighted how the pandemic led to higher levels of loneliness for both adults and adolescents [5,6,49]. In particular, female respondents reported higher levels of loneliness than their male counterparts both before [56] and during the pandemic [5,14]. However, it has also been suggested that the pandemic may have exacerbated this pre-existing gender difference, widening the gap between young men and young women in terms of loneliness [14]. Before the pandemic, young women had higher levels of loneliness than young men [56] and that result worsened in the current period—indeed, the gender difference has been accentuated, showing higher levels of loneliness among young women during pandemic [5,14]. For this reason, we have assumed the following hypotheses:

**H3.0.**  *The pandemic, and remote learning too, increased student’s perceived loneliness.*

**H3.1.**  *Before the pandemic, girls perceived more loneliness than boys.*

**H3.2.**  *During the pandemic, girls perceived an increased loneliness than boys.*

**H3.3.**  *The difference between boys’ perceived loneliness and girls’ perceived loneliness increased during the pandemic.*

This pandemic, as well as the social distancing measures, interrupted many social support networks [57]. This situation could have had an impact on Sense of Community; however, ICTs became a tool to keep connections intact: we know that, before the pandemic, distance learning actually had a positive effect on Sense of Community [58,59]. Therefore, we expected the following

**H4.0.**  *Sense of Community was only partially impacted by the pandemic.*

Lastly, scientific literature has highlighted a specific relation between loneliness and sense of community: even before the pandemic, a higher reported sense of community was correlated with lower loneliness [60,61]. For this reason, we assumed the following:

**H5.0.**  *There is a negative linear correlation between loneliness and sense of community, both in the pre-pandemic period and during the pandemic.*

## 2. Material and Methods

### 2.1. Materials

The online questionnaire has been structured into four main sections: socio-demographic questions, questions about the use of technology before and after COVID-19, and psychological questions about the perceived sense of loneliness and perceived sense of community, all of them before and after COVID-19. We started with socio-demographic questions, asking about the age of participants, their gender, and which grade they were in. We assessed the adolescents’ use of technologies before and after COVID-19 by using 14 ad hoc items (seven items were to measure the utilization before pandemic and 7 after) in which we have analyzed three different dimensions: “social dimension” (using technology to keep in touch with family/friends/class or to manage social network), “playful dimension” (using technology to play online games), and “work dimension” (using technology to study or stay updated on the news). We used a 5-point response format ranging from 1 (*strongly disagree*) to 5 (*strongly agree*). These 14 items have been produced by some focus groups formed by school psychologists, social psychologists, and psychologists of virtual environments. The experts involved in the process were asked to formulate items in relation to the three uses that the literature has underlined as particularly impacting ICT usage (i.e., recreational, social, and informational activities).

To measure the sense of loneliness, we used the 6-Item Loneliness Scale [62], a self-report questionnaire consisting of six items that capture three dimensions: to measure overall loneliness and emotional and social loneliness. The scale utilizes a 5-point response format ranging from 1 (“no”) to 5 (“yes!”). To assess the sense of community, we used The Classroom and School Community Inventory (CSI; [63]), a self-report questionnaire consisting of 20 items involving 10 classroom community items and 10 school community items which are divided into the classroom form and the school form, ranging from 1 (*strongly disagree*) to 5 (*strongly agree*). We used all three scales (use of technologies, Loneliness Scale, and CSI) in PRE (pre-COVID-19) and POST (during-COVID-19) forms to assess the students’ perceptions before and during COVID-19.

### 2.2. Sample and Procedure

Before proceeding with the recruitment, we carried out the power analysis to establish an adequate sample size for our research purposes. We relied on G*Power software to accomplish this procedure [64,65]. Power analysis allows scholars to define the sample size required to detect an effect of a given size with a given degree of confidence. For each type of statistical analysis, a power analysis should be performed, and the final sample size should be evaluated based on the power analysis that requires the largest sample size. The power analysis showed that a sample size of 787 adolescents would be enough to ensure a statistical power of 0.80 for assessing pre and post differences, assuming a small effect size (d = 0.10) and a significance level of 0.05. For testing gender-related differences, 164 individuals for the less numerous levels of the variable “gender” would be required to reach the same statistical power (i.e., 0.80), supposing a small–medium effect size (d = 0.25) and a gender ratio of 1:3.3 (which was derived from the gender ratio of the schools that were willing to join the data collection). Finally, for the correlation analysis, a sample of 782 would be needed to achieve a statistical power of 0.80 with a relatively small effect (r = 0.10). 

Given the exploratory nature of the work, the authors chose a nonprobability method based on the voluntary census to test their hypotheses. In these circumstances, studies based on voluntary participation can be considered satisfactory [66]. A total of 917 high school students (255 boys, 649 girls, 13 did not specify their gender) with an average age of 16.38 (standard deviation = 1.54) were recruited through their teachers and provided their informed consent. 

Participants attend different High School classes (in Italy, high school lasts for a period of 5 years): 14.8% were in 1st year, 23.7% were in 2nd year, 10% were in 3rd year, 28.1% were in 4th year, and 23.5% were in 5th year.

The questionnaires were administered to the participants during their remote-learning classes according to the Italian law’s requirements of privacy and informed consent (Law Decree DL-101/2018), EU regulation (2016/699), and APA guidelines. Overall, 33 students (3.5%) did not provide their informed consent and, thus, their data were not registered. 

### 2.3. Data Analysis

We first verified the preconditions necessary for testing our hypotheses. For all the continuous variables, we assessed normality (asymmetry and kurtosis values), homoscedasticity, and linearity. Then, we investigated pre and post differences through the paired Student’s *t*-test test, while for gender-related differences, we relied on Welch’s *t*-test since it performs better than Student’s *t*-test whenever sample sizes and variances are unequal between groups and gives the same result when sample sizes and variances are equal [67]. Finally, we used Pearson’s partial correlation to assess the relationship between sense of community and loneliness before and during the COVID-19 pandemic. 

## 3. Results

### Inferential Analysis

As the first step for inferential analyses, we investigated whether the COVID-19 pandemic and remote learning affected high school students’ use of technologies through paired sample *t*-tests. The results are shown in Table 1.

For the sake of clarity, we specify that a commonly used rule for Cohen’s d interpretation distinguishes small, medium, and large effect sizes for d values of 0.2, 0.5, and 0.8, respectively, based on benchmarks suggested by Cohen [68]. Nonetheless, this rule should not be interpreted rigidly [69].

Our respondents reported an overall increased use of technology due to the impact of the global pandemic and distance learning. In particular, there was a large increase related to studying and the need to keep in touch with class, followed by a nearly large increase in keeping in touch with friends and staying updated on the news. Keeping in touch with family and online gaming were described as having a small increase, but it is worth reiterating that even the variable with the lower reported increase (management of social networking sites) still saw some growth. As for the psychological variables included in our data collection, we observed a mild increase in the levels of Loneliness, as well as a slightly lowered Sense of Community: notably, the largest decrease can be seen in the School Sense of Community, while the Class Sense of Community, despite being statistically significant, declined in a negligible way. 

Subsequently, we investigated if there were some differences between young men and women in technology use, Sense of Community, and Loneliness both before and during the pandemic (Table 2).

We found a statistically significant gender-related distinction in the use of technologies. Specifically, girls appeared to use technology to keep in touch with their friends both before and during the COVID-19 pandemic much more than boys. Regarding the use of technologies to interact with one’s family, we observed that girls relied more on said technologies compared to boys during the pandemic. 

Moreover, the difference between genders regarding the use of technologies to stay updated on the news, which existed before the pandemic, disappeared in the current period.

In general, young women appeared to use technologies more than young men to manage social network accounts both before and during the pandemic. Nonetheless, this difference increased passing from the pre-pandemic period to the current one (i.e., from d_(PRE)_ = −0.26 to d_(POST)_ = −49). 

Furthermore, we found that young men used technologies to play online much more than young women, and this difference was stable in both the considered periods. 

Considering the use of technologies for study purposes, boys reported using them more than girls before the pandemic. Nonetheless, the difference between genders appeared to be reversed during the pandemic with the girls reporting a much greater use. 

No gender-related effects were found regarding the use of technologies to stay in touch with the class.

Another gender-related distinction emerged from the Loneliness Scale scores: young women reported feeling more lonely than young men both before and during the pandemic. Notably, the difference between young men and women deepened during the pandemic (i.e., Cohen’s d passed from a small one to a large one). 

Regarding participants’ Sense of Community, we found statistically significant gender differences, although with small effect sizes. Young men obtained higher scores in both classroom and school sense of community both before and during the pandemic. These gender differences remained stable in size in the two periods considered. 

To better highlight the interaction effects between gender and time, we first computed pre and during variation (i.e., Δ) for all the variables involved in our data collection. Subsequently, we investigated whether gender affected these scores. In this way, the gender differences that have remained constant between the two times considered (i.e., T1 = before the pandemic, T2 = during the pandemic) are not highlighted (i.e., absence of effect in both T1 and T2, similar difference detected in both T1 and T2). The results of this analysis are shown in Table 3. Female respondents modified their behavior from T1 to T2 to keep in touch with their family, study, and stay updated on the news more than male respondents. The analysis shown in Table Y provided indications regarding the direction of this modification: young women increased their use of technologies much more than young men for these specific purposes. Furthermore, compared to male respondents, young women had a larger change in Loneliness. Although young women had higher scores than young men in both T1 and T2, the difference became much wider in T2. Since gender appeared to affect both loneliness and sense of community scores in the previous analyses with non-negligible effect size, we relied on a partial correlation technique to investigate how loneliness and sense of community were related to one another. Partial correlation allowed controlling for gender-related effects and thus obtaining an effect size without the confounding effect of the gender variable.

In Table 4, we further analyzed the partial correlation between the SOC and Loneliness dimensions controlled for gender. Loneliness PRE seems to influence Loneliness DURING with an effect size of a mere 17%. Concerning SOC, on the other hand, the level referred before the pandemic seems to influence with a greater effect size the value reached at T2; more specifically, the effect size is 38–48% depending on the type of SOC examined (i.e., Class or School). Moreover, by investigating the inter-correlations between SOC variables at different times, we came up with two different findings: SOC Class at T1 was related to SOC School at T2 (with a common variance of 21%), and SOC School at T1 was related to SOC Class at T2 (with a common variance of 25%). By analyzing the inter-correlations between the two SOC dimensions (i.e., School and Class) referring to the same period of time, we observed that they correlated 0.71 both before and during the COVID-19 pandemic. 

We observed a relatively large correlation between Loneliness PRE and SOC PRE, both with Class and School. Moreover, Loneliness PRE levels were associated with SOC DURING levels, both for Class and School, but with a smaller effect size. Finally, Loneliness POST seemed to be correlated with SOC PRE levels with a small/typical effect size, while Loneliness appeared to have a relatively large relation to SOC DURING. For the sake of clarity, the Pearson r coefficient can be interpreted considering values of 0.10, 0.20, and 0.30 as relatively small, typical, and large [70], respectively, when investigating the relationship between two different variables (i.e., that do not belong to the same construct). 

## 4. Discussion

The current pandemic has had a disruptive effect on people all around the world, including teenage students [2]. Unlike other communities, students had the opportunity to keep in touch with their social contacts through remote-learning [7] and personal ICT social opportunities [5,14,29]. 

In the present work, we showed that adolescents’ use of technology to study (H1.1), keep in touch with friends and classmates (H1.2), keep up with the news (H1.3), and play games online (H1.4), has changed, and they seem to use these technologies much more than they did before.

We have also seen how the use of technology was influenced by gender (H2.0). Girls, before and during the pandemic, used ICTs to keep in touch with friends and families more than boys (H2.1), while boys used ICTs to play games online more than girls before and during the pandemic (H2.2). Some gender differences in ICT usage were reduced (e.g., to keep with the news, partially supporting H2.3) or reversed (e.g., to study, as girls started using ICTs more than boys for this purpose).

The pandemic has also heightened adolescents’ sense of loneliness, thus confirming H3.0. This result also appeared to be affected by gender, with girls seemingly being mostly affected (H3.2). In accordance with the literature [56], we hypothesized and found that female respondents reported higher levels of loneliness even before the pandemic started (H3.1) and that COVID-19 only widened the imbalance between male and female respondents in terms of perceived loneliness (H3.3).

SOC, instead, was only modestly impacted by the pandemic, in line with our hypothesis (H4.0). This probably also happened thanks to ICTs, which helped maintain relationships with classmates and schoolmates as already reported in some works conducted before the pandemic [59,63].

Finally, we confirmed the well-known relationship between SOC and Loneliness both in the period before and during the COVID-19 pandemic (H5.0). The discrepancy in the strength of the relationship between SOC and Loneliness in pre-pandemic and pandemic periods appeared small (r ≃ 0.05) and may indicate that school and class-related SOCs contributed slightly more to defining adolescents’ loneliness when other sources of belongingness were not challenged.

Overall, our work suggested that the impact of the COVID-19 pandemic on Italian adolescents could be related to their modification of ICT usage for remote learning purposes. Although a substantial lowering of loneliness levels is undeniable (and it is more pronounced in female respondents), high school students’ sense of community remained almost unchanged even though they could not interact face-to-face with each other for months. These results appeared consistent with SIT [21], SCT [22,23], and Need to Belong theory [71]. Indeed, the students’ identification with school and class environments was already established prior to the pandemic and ICTs appeared to provide them with enough opportunity to socially engage. However, this compensation was not totally possible for any other source of social belonging and thus loneliness was affected.

Nevertheless, several limitations of this study need to be addressed. First of all, the study is based on retrospective perceptions of adolescents’ use of technology, sense of community, and loneliness. Thus, recall bias, implicit theory of change, and present state effect may have affected our results [72,73,74,75,76,77,78]. For instance, participants may remember their pre-pandemic state as better than it actually was. The use of self-reported measures could have introduced some biases due to the well-known tendency of people to provide socially acceptable answers rather than being truthful (i.e., honesty) and their limited capability of assessing themselves accurately (i.e., introspective ability), especially retrospectively [79,80]. Moreover, given our study design, no inferences regarding causality can be drawn. Thus, future studies using different research designs are required. Another limitation associated with our study is that the increase in familywise error rate across the reported statistical analyses was not controlled. Of course, in order to prove that our study results are due to ICT usage and not just by young people’s higher adaptability, future research should try to compare these results with those obtained from other populations where the use of technology was not so common before the pandemic. The role of ICTs and more generally of the Internet in emergency scenarios like the pandemic is far from being fully understood. Indeed, the web can offer not only young adults and students an opportunity to cultivate their general sense of belonging but also to receive social support [81,82], which could lead to better adaptation to emergency situations [83,84,85]. Future research should also clarify how loneliness varies in remote learning and blended learning conditions outside the current pandemic. This would allow scholars to have a more complete idea of the psychological repercussions related to remote learning [14,86] in a world where the distinction between real and virtual is increasingly nuanced [87,88,89,90]. Based on what emerged from our research, the importance of remote learning, a process that had already begun several years ago (e.g., telematic universities, e-learning platforms), but which had a decisive turning point in the pandemic period, has been known. The increasing adoption of ICTs has proven to be a valuable aid both in coping with the negative consequences of the pandemic [91], and in enhancing existing learning tools. This method certainly does not prove to be exempt from important limits; however, given the power shown above all in the pandemic period, the importance of improving this method emerges, especially in view of a growing and future use of these methods.

## 5. Conclusions

In conclusion, the pandemic greatly exacerbated the loneliness of Italian high-school students (especially in young women), but not their sense of community. Indeed, ICTs plausibly allowed adolescents to foster their sense of community despite the current COVID-19 pandemic situation that, due to lockdown measures and remote learning, nearly resembles the dystopic condition described by Isaac Asimov for Solaria inhabitants who communicate almost entirely through sci-fi ICTs:

“They live completely apart and never see one another except under the most extraordinary circumstances”.

-*Isaac Asimov*, The Naked Sun [92]

## Figures and Tables

**Table 1:** Paired Student’s *t*-test for assessing differences between the pre-pandemic and current period.

Variable	PRE DURING	M	s.d.	*t*	df	*p.*	Cohen’s d
To keep in touch with my friends	PRE	3.89	0.98				
	DURING	4.56	0.77	−21.34	916	<0.001	0.71
To keep in touch with my class	PRE	3.23	1.09				
	DURING	4.19	0.97	−25.55	916	<0.001	0.86
To keep in touch with my family	PRE	2.93	1.31				
	DURING	3.50	1.28	−14.92	916	<0.001	0.49
To play online	PRE	2.65	1.39				
	DURING	2.93	1.47	−8.63	916	<0.001	0.28
To study	PRE	2.80	1.18				
	DURING	4.51	0.79	−38.66	916	<0.001	1.28
To stay updated on the news	PRE	3.44	1.22				
	DURING	4.11	0.96	−20.72	916	<0.001	0.69
To manage my social network accounts	PRE	4.16	1.08				
	DURING	4.26	1.06	−4.67	916	<0.001	0.16
Loneliness	PRE	14.20	4.62				
	DURING	17.73	5.00	−3.84	916	<0.001	0.74
Sense of Community (Class)	PRE	31.72	7.86				
	DURING	31.14	8.17	2.58	916	0.01	0.08
Sense of Community (School)	PRE	33.14	7.38				
	DURING	31.48	7.88	8.42	916	<0.001	0.28

**Table 2:** Gender-related differences in technology use, SOC, and Loneliness.

Variable	PRE- DURING	Boys	Girls	*t*	df	*p.*	Cohen’s d
		M(s.d)	M(s.d)				
To keep in touch with my friends	PRE	3.73 (1.00)	3.96 (0.95)	−3.23	443.73	<0.001	−0.24
	DURING	4.30 (0.90)	4.66 (0.68)	−5.73	373.40	<0.001	−0.45
To keep in touch with my class	PRE	3.27 (1.11)	3.23 (1.08)	0.40	452.55	0.69	
	DURING	4.18 (1.00)	4.20 (0.95)	−0.21	442.70	0.83	
To keep in touch with my family	PRE	2.85 (1.28)	2.98 (1.32)	−1.32	476.68	0.19	
	DURING	3.16 (1.32)	3.64 (1.25)	−4.98	442.59	<0.001	−0.37
To play online	PRE	3.43 (1.37)	2.31 (1.26)	11.28	432.82	<0.001	0.85
	DURING	3.70 (1.27)	2.61 (1.42)	11.23	514.95	<0.001	0.81
To study	PRE	3.02 (1.17)	2.71 (1.17)	−3.68	466.22	<0.001	0.26
	DURING	4.29 (0.89)	4.60 (0.74)	−4.89	398.03	<0.001	−0.38
To stay updated on the news	PRE	3.56 (1.14)	3.39 (1.25)	1.99	507.08	0.047	0.14
	DURING	4.04 (1.02)	4.13 (0.94)	−1.21	432.74	0.227	
To maintain my social network accounts	PRE	3.85 (1.23)	4.28 (0.98)	−5.08	389.44	<0.001	−0.26
	DURING	3.88 (1.22)	4.40 (0.95)	−6.10	382.64	<0.001	−0.49
Loneliness	PRE	13.02 (4.12)	14.56 (4.69)	−4.86	525.40	<0.001	−0.35
	DURING	15.23 (4.79)	18.70 (4.76)	−9.82	462.45	<0.001	−0.73
Sense of Community (Class)	PRE	33.92 (7.47)	30.97 (7.84)	5.26	485.76	<0.001	0.38
	DURING	33.24 (8.30)	30.34 (7.98)	4.77	449.07	<0.001	0.35
Sense of Community (School)	PRE	34.02 (6.95)	32.95 (7.46)	2.05	496.09	0.041	0.15
	DURING	32.69 (7.80)	31.10 (7.82)	2.75	465.51	0.006	0.20

Note: N_(boys)_ = 255; N_(girls)_ = 648.

**Table 3:** Gender-related differences regarding the pre and post variations in technology use, SOC, and Loneliness.

Variable	Gender	M	s.d.	*t*	df	*p.*	Cohen’s d
Δ To keep in touch with my friends	Boys	0.57	0.94				
	Girls	0.69	0.93	−1.76	459.61	0.08	
Δ To keep in touch with my class	B	0.91	1.21				
	G	0.96	1.09	−0.55	424.32	0.58	
Δ To keep in touch with my family	B	0.31	1.03				
	G	0.66	1.19	−4.42	534.78	<0.001	−0.31
Δ To play online	B	0.27	0.91				
	G	0.29	1.03	−0.33	517.84	0.74	
Δ To study	B	1.27	1.34				
	G	1.89	1.30	−6.37	452.71	<0.001	−0.47
Δ To stay updated on the news	B	0.49	0.93				
	G	0.75	0.99	−3.74	493.93	<0.001	−0.27
Δ To manage my social network accounts	B	0.03	0.71				
	G	0.12	0.59	−1.64	400.29	0.10	
Δ Loneliness	B	2.21	4.03				
	G	4.14	4.91	−6.07	561.81	<0.001	−0.43
Δ Sense of Community (Class)	B	−0.68	5.52				
	G	−0.63	7.31	−0.12	610.86	0.91	
Δ Sense of Community (School)	B	−1.34	5.05				
	G	−1.85	6.24	1.29	570.13	0.20	

Note: N_(boys)_ = 255; N_(girls)_ = 648; Δ = pre-post variation.

**Table 4:** Partial correlation of SOC and Loneliness (controlled for gender).

Variable	1	2	3	4	5	6
1. PRE-Loneliness	1	0.49 ***	−0.33 ***	−0.40 ***	−0.21 ***	−0.28 ***
2. DURING-Loneliness	0.49 ***	1	−0.12 ***	−0.21 ***	−0.31 ***	−0.35 ***
3. PRE-SOC (Class)	−0.33 ***	−0.12 ***	1	0.71 ***	0.62 ***	0.46 ***
4. PRE-SOC (School)	−0.40 ***	−0.21 ***	0.71 ***	1	0.50 ***	0.69 ***
5. DURING-SOC (Class)	−0.21 ***	−0.31 ***	0.62 ***	0.50 ***	1	0.71 ***
6. DURING-SOC (School)	−0.28 ***	−0.35 ***	0.46 ***	0.69 ***	0.71 ***	1

Note: N = 917; df = 914; *** *p* < 0.001.

## Data Availability

The data presented in this study are available on request from the corresponding author.
